# Impact of a medical supply bulk-buy program on treatment of patients with coronary artery disease in China: A single-center study

**DOI:** 10.1371/journal.pone.0285528

**Published:** 2023-05-17

**Authors:** Xinxing Song, Yanzhuo Ma, Zhiwen Li, Xiaoye Wang, Lingfeng Kong, Gang Wang, Yuhong Peng, Leisheng Ru

**Affiliations:** Department of Cardiology, Bethune International Peace Hospital, Shijiazhuang, Hebei, China; University of Hull, UNITED KINGDOM

## Abstract

**Background:**

The Chinese government recently introduced a program to buy medical supplies in bulk to reduce the patient cost burden. For patients undergoing percutaneous coronary intervention (PCI), little is known about the effect on outcomes of this bulk-buy program.

**Aims:**

This study investigated whether the bulk-buy program to decrease the price of stents used in PCI affected clinical decision-making and outcomes.

**Methods:**

This single-center study enrolled patients undergoing PCI from January 2020–December 2021. Prices decreased for stents on January 1, 2021, and balloons on March 1, 2021. Patients were grouped by surgical year as either before (2020) or after (2021) policy implementation. All clinical data were collected. To examine whether clinical decision-making for PCI was affected by the bulk-buy program, procedure appropriateness was analyzed using the 2017 appropriate use criteria (AUC). To assess outcomes, the rates of major adverse cardiac and cerebrovascular events (MACCE) and complications were compared between groups.

**Results:**

Study participants were 601 patients in 2020 (before bulk buying) and 699 patients in 2021 (after bulk buying). Results of analysis by AUC for procedure appropriateness were 74.5% appropriate, 21.6% may be appropriate, and 3.8% rarely appropriate in 2020, with no differences for patients who underwent PCI in 2021. Between-group comparisons showed MACCE rates of 0.5% in 2020 and 0.6% in 2021, whereas complication rates were 5.5% and 5.7%, respectively. No statistically significant differences were found between groups (p > 0.05).

**Conclusion:**

The bulk-buy program did not impact physician clinical decision-making or surgical outcomes for patients undergoing PCI.

## Introduction

Although many efforts have recently been introduced to improve people’s health in China, the vast territory and unbalanced economic development have limited these efforts, especially in patients with cardiovascular disease [1–3]. As one of the most serious diseases threatening human health, coronary heart disease (CHD) consumes vast medical resources [4, 5]. One of the key treatments for CHD is percutaneous transluminal coronary intervention (PCI), however, this treatment is costly because of the expensive stents and balloons used for this procedure, which imposes a high financial burden for patients and society. Another valid strategy for coronary artery disease is coronary artery bypass grafting (CABG), while in China, patients prefer PCI and drug therapy because of the high cost and invasiveness [6, 7]. To overcome this obstacle, the Chinese government launched a series of national healthcare reforms, one of which is the *bulk-buy program*. Under this program, the price of drugs and medical consumables listed in the procurement catalog declined significantly. Specifically for PCI, the prices for stents decreased from $2000 U.S. dollars to $100 U.S. dollars and for balloons decreased from $800 U.S. dollars to $60 U.S. dollars from January 1, 2021, to March 1, 20201, respectively.

Despite these significant cost savings, while some have welcomed the move, others have expressed concerns that this bulk-buy program may affect product safety or lead to stent abuse. The impact of this bulk-buy program on the treatment of CHD is not yet known. To address this concern for patients with coronary artery disease undergoing treatment with PCI, our study sought to assess the impact of the bulk-buy program on this population by collecting clinical data before and after program implementation, specifically to determine any effects of the program on physician clinical decision-making and surgical outcomes for patients.

## Methods

### Study design

This retrospective, single-center study used the 2017 Appropriate Use Criteria (AUC) to analyze the appropriateness of the PCI procedures performed before and after implementation of the bulk-buy program, which would explain whether the implementation of bulk buying influenced practice patterns and decision-making among clinicians. The rates of MACCEs and complications associated with PCI were also analyzed to determine whether the bulk-buy program influenced procedure safety.

### Study groups

From January 1 to December 31, 2020, a total of 1356 consecutive patients who underwent PCI at our hospital were retrospectively enrolled in Group 2020. After introduction of the bulk-buy program, from January 1 to December 31, 2021, 1548 consecutive patients who underwent PCI were included as Group 2021. We determined the exclusion criteria based on a review of patient medical records, and the following patient groups were not included: 731 patients (25.2%) with missing background data, and 873 patients (30.1%) who underwent PCI for emergency indications, such as unstable angina and acute myocardial infarction, including Canadian Cardiovascular Society class 4. The remaining 1300 patients were included for analysis.

Due to the retrospective nature of the study, informed consent was waived. Our study was approved by the Ethics Committee of Bethune International Peaceful Hospital (approval number 2022-KY-4). This study was conducted in accordance with the principles of the Declaration of Helsinki.

### Data collection

Well-trained resident physicians collected clinical information with a focus on data used in the 2017 AUC, including: 1) clinical presentation; 2) Canadian Cardiovascular Society (CCS) class; and 3) number of anti-ischemic medical treatments. Other AUC-related data, including patient demographics and clinical and procedural characteristics, were collected through medical records.

### Study definition

The key variables defined by the 2017 AUC for coronary revascularization in patients with stable ischemic heart disease were used to classify the “appropriateness” of the procedure. Briefly, classification of vessel disease was based on previous literature criteria [8] as follows: whether proximal left anterior descending (LAD) artery or proximal left dominant left circumflex artery was implicated, anginal symptoms, bifurcation involvement, concurrent multi-vessel disease, SYNTAX score, noninvasive risk stratification, and the number of antianginal drugs at discharge.

Patients were stratified as high noninvasive risk or intermediate noninvasive risk if the patient fulfilled at least one of the following standards: left ventricular ejection fraction <50%, and a positive stress electrocardiography (ECG) test result. A lesion was considered as intermediate complexity or high complexity if patient had an unprotected left main coronary artery or three-vessel disease and the SYNTAX score was >22. When determining the invasive risk stratification for this study, the 909 (70%) patients who did not have stress ECG results available or were not registered for an alternative noninvasive stress test were considered to have negative results on stress ECG. Successful PCI was defined as complete passage of both the guidewire and balloon through the lesion, successful dilation of the occluded artery, and restoration of antegrade flow (thrombolysis in myocardial infarction grade flow score of 3) with <50% residual stenosis at the time of final angiography. In-hospital major adverse cardiac and cerebrovascular events consisted of all cause-death, nonfatal myocardial infarction, emergent target vessel revascularization with PCI or coronary artery bypass grafting, stroke, and significant angina. Myocardial infarction was defined according to the Fourth Universal Definition of Myocardial Infarction Guidelines. Complications associated with the procedure were defined as bradycardia, coronary perforation with or without cardiac tamponade, coronary dissection, entrapped equipment, and no reflow.

### Statistical analysis

Continuous variables were indicated as mean ± standard deviation. Categorical values were shown as percentages. Continuous data were compared using unpaired student t-test for parametric data and the Mann-Whitney U-test for non-parametric data. Categorical variables were expressed as counts (percentages) and analyzed by using the rank sum or Fisher exact test, as appropriate. A value of p < 0.05 indicated statistical significance, and in all analyses the p values were two-sided. Analyses were conducted using SPSS version 21.0 (SPSS, Chicago, Illinois, USA).

## Results

### Patients in the analysis

Percutaneous coronary intervention was performed for 1356 patients from January–December 2020 and for 1548 patients from January–December 2021. Based on the exclusion criteria, 755 patients were excluded from Group 2020 and 849 from Group 2021; therefore, a total of 1300 patients were enrolled in this study: 601 patients in Group 2020 and 699 patients in Group 2021. Subsequently, the clinical data for all 1300 patients were analyzed.

**Fig 1:**
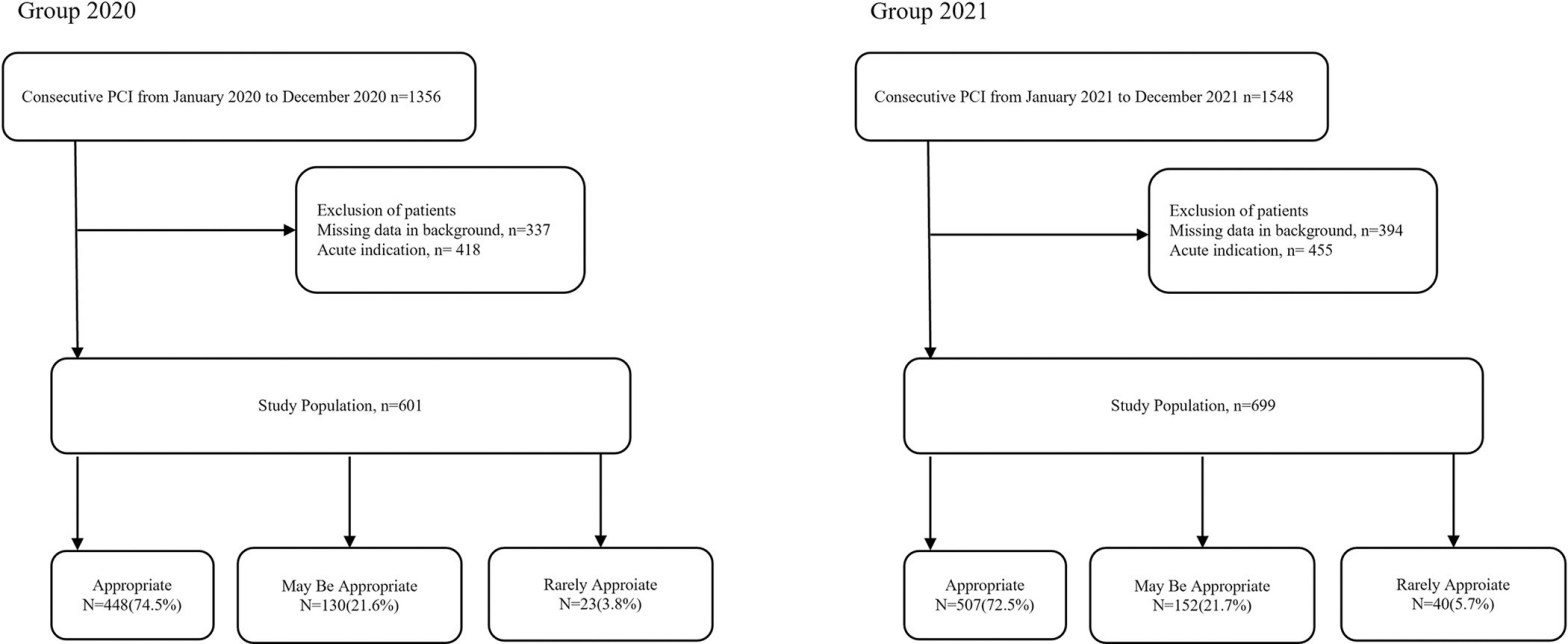
Study flowchart. PCI, percutaneous coronary intervention.

For the PCI procedures in the 601 Group 2020 patients, 448 (74.5%) were classified as “appropriate,” 130 (21.6%) as “may be appropriate,” and 23 (3.8%) as “rarely appropriate” using the 2017 AUC. For the PCI procedures in the 699 Group 2021 patients, 507 (72.5%) were classified as “appropriate,” 152 (21.7%) as may be “appropriate,” and 40 (5.7%) as “rarely appropriate” (Fig 1).

### Baseline clinical characteristics

Data for the patients groups are presented in Table 1. Cardiovascular risk factors were very prevalent—64.2% of patients had hypertension and 41.7% had dyslipidemia. Compared with Group 2020, the patients in Group 2021 had a higher prevalence of peripheral arterial disease, a higher CCS class II, increased anticoagulant use, a higher number of multiple coronary lesions, and a higher degree of proximal LAD involvement. Notably, however, the average hospital length of stay and average hospital costs were lower in the Group 2021 patients. In addition, more drug-coated balloons were used in 2021 versus 2020 (110 vs. 32; p<0.05). Moreover, intravascular ultrasound was performed more often in 2021.

**Table 1:** Baseline characteristics of patients undergoing PCI according to appropriateness categories.

	2020 n = 601	2021 n = 699	P value
Age, years	61.6(10.5)	62.1(10.5)	0.39
LVEF,%	60.5(8.5)	61.2(8.4)	0.14
eGFR, ml/min/1.73m^2^	90.9(18.7)	91.0(16.1)	0.92
Male sex	425(70.7)	512(73.2)	0.31
Current smoker	94(15.6)	125(17.9)	0.28
Hypertension	388(64.6)	447(63.9)	0.82
Dyslipodemia	252(41.9)	290(41.5)	0.87
Diabetes	206(34.3)	216(30.9)	0.19
History MI	129(21.5)	127(18.2)	0.91
Prior PCI	197(32.8)	231(33.0)	0.92
Deripheral artery disease	95(15.8)	123(17.6)	0.03
Anticoagulants	6(1.0)	11(1.6)	0.001
Ace inhibitors	92(15.3)	102(14.6)	0.72
ARBs	183(30.4)	205(29.3)	0.66
Stains	594(99.0)	692(99.0)	0.78
B-blockers	456(75.9)	520(74.4)	0.54
Calcium channel blocker	244(40.6)	279(39.9)	0.80
Nitrates	447(74.4)	536(76.7)	0.33
Key variables			
anginal symptoms			<0.05
no symptoms	19(3.2)	16(2.3)	
CCS class I	238(39.6)	181(25.9)	
CCS class II	283(47.1)	440(62.9)	
CCS class III	61(10.1)	62(8.9)	
High or intermediate risk in Non-invasive tests	506(84.2)	561(80.2)	0.07
No. of antianginal drugs			0.45
0	28(4.7)	36(5.1)	
1	198(32.9)	225(32.2)	
2	324(53.9)	356(50.9)	
3	51(8.5)	83(11.8)	
No. of diseased vessels			0.007
1	252(41.9)	239(34.2)	
2	228(38.0)	294(42.1)	
3	121(20.1)	164(23.7)	
LM involvement	75(12.5)	99(14.2)	0.37
Proximal LAD involvement	274(45.6)	391(55.9)	0.002
CTO lesions	212(35.3)	253(36.2)	0.73
Average hospital stay	8.0(3.2)	7.5(2.9)	0.003
Average hospitalization cost($)	8592.3(855.5)	5362.6(2811.7)	<0.001
No. of stents used			0.07
0*	26(4.4)	64(9.3)	
1	286(48.8)	323(47.1)	
2	171(29.2)	183(26.7)	
≥3	103(19.6)	116(16.9)	
Procedure success	567(94.3)	667(95.4)	0.38
No. of drug coated balloons used	32(5.3)	110(15.7)	<0.05
No. of IVUS used	168	268	

Continuous variables (Age, LVEF, and eGFR) are represented as mean (SD), and categorical variables as n (%).

* Failure procedure were exclude. ACE, angiotensin converting enzyme; ARB, angiotensin receptor blocker; CCS, Canadian Cardiovascular Society; eGFR, estimated glomerular filtration rate; LAD, left anterior descending artery; LM, left main trunk; LVEF, left ventricular ejection fraction; MI, myocardial infarction; PCI, percutaneous coronary intervention; IVUS, intravascular ultrasound.

### Procedure appropriateness

Table 2 shows that the rates of appropriate, may be appropriate, and rarely appropriate procedures in 2020 and 2021 were very similar, with no significant statistical differences. Notably, the proportion of rarely appropriate procedures was very low—3.9% in 2020 and 5.7% in 2021. Overall, the proportion of patients who underwent as appropriate PCI reached 73.5%.

**Table 2:** Destitution of appropriateness related to the procedure.

	2020	2021	P value
Appropriate	448(74.5)	507(72.5)	0.12
May Be Appropriate	130(21.6)	152(21.7)	0.96
Rarely Appropriate	23(3.9)	40(5.7)	0.11

Data are presented as n (%).

### In-hospital MACCEs and complications

A brief summary of in-hospital MACCEs and complications associated with PCI are shown in Table 3. In Group 2020, the in-hospital mortality cases were 2 patients who died suddenly because of suspected in-stent thrombosis and 1 patient who had a stroke. In Group 2021, 3 patients died in hospital; 1 patient had a target vessel revascularization, and 1 patient experienced a nonfatal myocardial infarction. No significant statistical differences were observed between the incidence of MACCEs in 2020 and 2021. Likewise, the rates of complications associated with PCI did not differ between 2020 and 2021.

**Table 3:** In-hospital MACCE and complications related to PCI.

	2020(n = 601)	2021 (n = 701)	P value
MACCE			0.73
All cause-death	2(0.3)	3(0.4)	
Non-fatal MI	0(N)	1(0.1)	
Target Vessel Revascularization	0(N)	1(0.1)	
Significant angina	0(N)	0(N)	
Stroke	1(0.2)	0(N)	
Complications* related to PCI	33(5.5)	40(5.7)	0.86

Data were presented as n (%). Complications in our study included bradycardia, coronary dissection or perforation, slow-no-reflow and gastrointestinal bleeding. MACCE = major adverse cardiac and cerebrovascular event(s); MI = myocardial infarction; other abbreviation as in Table 1.

## Discussion

One of the leading causes of death in both developed and developing countries such as China is CHD [9]. Optimal medical therapy, PCI, and coronary artery bypass grafting are the main treatments for CHD [10–12]. Depending on different types of lesions, different approaches to therapy are used [13–15]. In China, patients tend to prefer PCI as a main choice of treatment for coronary artery disease. With the increasing use of PCI in China, the cost for the stent places a financial burden on the patients and society. To solve this problem, China recently launched a series of national healthcare reforms, including the bulk-buy program for drugs and medical consumables.

In brief, in this bulk-buy program the government procures a particular drug or medical device in bulk by bidding to obtain a reduced price; once the government has procured a purchased item in bulk at a low price, it then sells the item to patients at the same price. Centralized procurement of coronary stents and balloon is expected to decrease prices in China by at least 90% and save patients billions of dollars a year. Despite these cost savings, however, many people express concerns regarding whether the bulk-buy program could affect procedure safety. It is unclear whether the reduction in price of a stent or balloon will influence clinical decision-making by an interventional cardiologist, leading to overuse of stents, or if it will affect product safety. To date, this policy has been carried out for 1 full year beginning on January 1, 2021. In this context, we conducted this study to address several of these important questions.

Our data showed that, owing to the reduction in stent/balloon costs, the average hospitalization cost was significantly lower after healthcare reform (8592.3$ vs. 5362.6$; p = 0.001) (**Table 1**). Although slightly fewer patients were treated with PCI in 2020 than in 2021, this phenomenon can be explained by the isolation policy during the coronavirus disease 2019 pandemic.

Before 2021, when physicians in our center were managing patients with lesions that required in-stent restenosis, they based their clinical decision-making on considerations of the costs for hospitalization and the common options of plain balloon angioplasty or implantation of drug-eluting stents, although—despite being costly—drug-coated balloons are generally the best option. With the removal of the cost barrier for use of an expensive stent/balloon, some high-priced treatments became more affordable, and physicians were able to choose the best treatment option according to the lesion type. Therefore, it was not surprising that drug-coated balloons and intravascular ultrasound were used more frequently in 2021.

The 2017 AUC is used to optimize the quality of clinical practice and provide a framework for the assessment of practice patterns, which will hopefully improve physician clinical decision-making. Recently, the United States, Europe, and Japan have released their own AUC [16–18]. In our study, the 2017 American Heart Association AUC was used to determine whether the cardiologist’s decision was influenced by the bulk-buy program. Data show that before the bulk-buy program in 2020, the percentages of “appropriate,” “may be appropriate,” and “rarely appropriate” were 74.5%, 21.6%, and 3.9%, respectively, whereas after bulk-buy program in 2021 the percentages were 72.5%, 21.7%, and 5.7%, respectively. No statistically significant differences were detected among these data—these results indicate that clinic decision-making was not affected by the bulk-buy program. Compared with the research by Hess et al. [19], our rate of appropriate PCI is notably higher(73.5% vs. 29.8%). The percentage of appropriate PCI can range from 25.1% to 47.1% in different studies [8, 19–21]. Regional variations, such as Asia, North America or Europe, and different types of lesions, such as percentage of CTO lesions vs. non-CTO lesions, may be the main contributors to this difference.

Another concern is that the falling price of medical consumables may made less the quality of the stent/balloon quality and increase treatment risks or complication rates. To elucidate the answer to this question, we statistically calculated the rates of MACCE and complications of PCI in 2020 and 2021. Our results showed no statistically significant differences in the overall MACCE rate between 2020 and 2021, and no statistically significant difference in the incidence of overall complications was observed. Our findings for the MACCE rate are consistent with those of previous studies [22, 23]. However, our result for the rates of complications is definitely higher. It should be noted that the most common complication is coronary perforation that occurs in patients with CTO who undergo PCI. By comparing the incidence of MACCE and complications in 2020 and 2021, the falling price of medical consumables did not increase complication rates for PCI in this study. Our results help to dispel concerns about the declining quality of medical consumables.

## Limitations

This research is a retrospective single-center study. Our center is dedicated to the interventional treatment of patients with complex coronary lesions, so the proportion of three-branch lesions, left main lesions, and CTO lesions is relatively high and may not be representative of the actual situation in most cardiac centers. Therefore, the results of this study must be evaluated with caution. Thirdly, long-term follow-up results are needed to assess the long-term impact of the bulk-buy policy. Lastly, only stress ECG was included as the diagnostic test in our study, and stress echocardiography, stress magnetic resonance imaging, or single-photon emission-computed tomography were not included, and this lack of information could lead to bias.

## Conclusion

To our best knowledge, this study is the first to investigate effect of a bulk-buy program on PCI. Our results show that the bulk-buy program has significantly reduced the cost of the therapy for CHD, alleviates the patient financial burden, and do not impact clinical decision-making and outcomes for PCI. Therefore, more drugs and medical consumables should be included in the bulk-buy program. Our study shows bulk-buy program is effective in reducing patient burden without causing changes in medical outcomes. In other words, this policy effectively reduces the burden on patients and government health insurance while ensuring the effectiveness of treatment. This study may provide an important perspective for healthcare reform in other countries. Bulk-buy program appears to be feasible to be extended to other countries or regions.

## Supporting information

S1 File Baseline characteristics analyzed in this study. (XLS)

## Abbreviations

AUC: Appropriate Use Criteria
CABG: Coronary artery bypass grafting
CCS: Canadian Cardiovascular Society
CTO: Chronic total occlusion
CHD: Coronary heart disease
ECG: Electrocardiography
LAD: Left anterior descending
MACCE: Major adverse cardiac and cerebrovascular events
PCI: Percutaneous coronary intervention
